# Inverted duplicate DNA sequences increase translocation rates through sequencing nanopores resulting in reduced base calling accuracy

**DOI:** 10.1093/nar/gkaa206

**Published:** 2020-04-07

**Authors:** Pieter Spealman, Jaden Burrell, David Gresham

**Affiliations:** Center for Genomics and Systems Biology, Department of Biology, New York University, New York, NY 10003, USA

## Abstract

Inverted duplicated DNA sequences are a common feature of structural variants (SVs) and copy number variants (CNVs). Analysis of CNVs containing inverted duplicated DNA sequences using nanopore sequencing identified recurrent aberrant behavior characterized by low confidence, incorrect and missed base calls. Inverted duplicate DNA sequences in both yeast and human samples were observed to have systematic elevation in the electrical current detected at the nanopore, increased translocation rates and decreased sampling rates. The coincidence of inverted duplicated DNA sequences with dramatically reduced sequencing accuracy and an increased translocation rate suggests that secondary DNA structures may interfere with the dynamics of transit of the DNA through the nanopore.

## INTRODUCTION

Advances in DNA sequencing have led to a greater understanding of the role structural variants play in disease (1–3) and evolution (4). However, high-throughput short read DNA sequencing faces intrinsic challenges in identifying complex structural variants. A variety of computational methods use sequence read depth, split and discordant reads to identify structural variants from short read DNA sequencing. However, these methods are uninformative in low complexity and repetitive regions of the genome (2,5,6). Long-read DNA sequencing can potentially overcome these technical limitations by generating reads of 10–100 kb in length that enable unambiguous resolution of contiguous DNA sequences (7–9). Nanopore sequencing, developed by Oxford-Nanopore Technologies (ONT), entails a sequencing pore that translocates DNA or RNA across a membrane and sensors that measure changes in ionic current during the translocation. The electrical signals are subsequently converted into base-called sequences using a machine-learning base-caller (8,10). The very long sequence reads that can be generated using this technology has proven invaluable for identifying many types of structural variants (SVs), such as translocations, tandem duplications and simple inversions which are difficult to identify using short-read sequencing alone (11,12).

**Figure 1.**
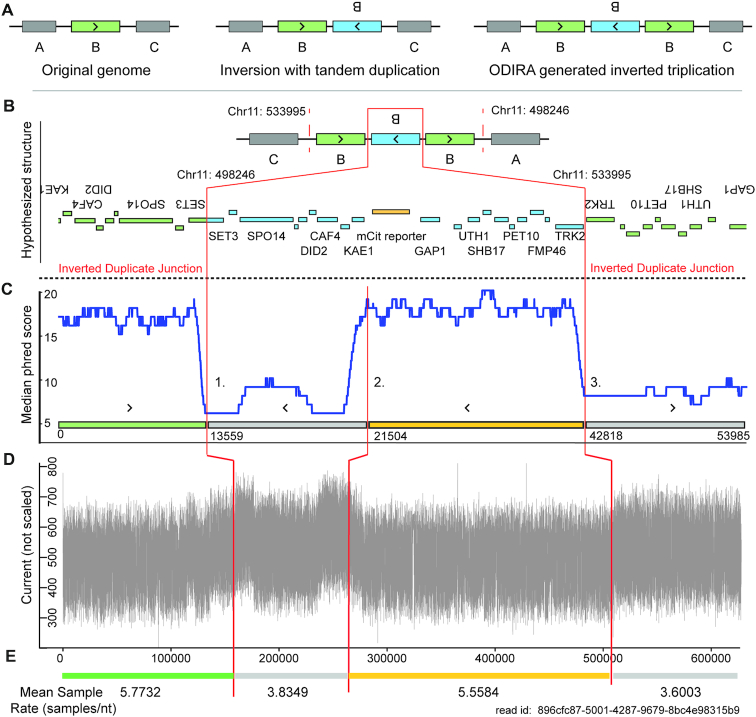
Schematic of a long-read sequence generated using nanopore sequencing that spans two inverted duplicate junctions. This example shows a single read spanning a known CNV containing a triplication that comprises two inverted duplications (**A**). The hypothesized structure based on the alignment of Illumina sequencing reads to the reference genome (16) (**B**). The predicted inverted duplicate junctions (16) are denoted with two vertical red lines. A rolling 1000-base window median phred score is calculated for the read (**C**). The low-phred scoring regions begin at the predicted junction (points 1, 3) and recovers after a distance similar to the length of the preceding normal quality region (2). An analysis of the raw current generated by the nanopore during the translocation of the DNA molecule shows a substantial uplift in current co-occurs around the low-phred scoring regions and the junction between inverted duplicated sequence (**D**). We find that the mean sample rate per nucleotide for these uplifted regions is also significantly lower than that observed for the normal quality region **(E)**.

Inverted duplicated DNA sequences are found in a variety of different classes of copy-number variants (CNVs) in which duplicated regions are oriented in opposite directions with a junction between them (Figure 1A) (13). Inverted duplications can include several distinct CNVs including inverted tandem duplications as well as more complex CNVs such as duplicate–triplicate/inversion–duplicate (DUP–TRP/INV–DUP) (3), duplicate–normal–duplicate (DUP–NML–DUP) (14) and origin dependent inverted repeat amplification (ODIRA), which result in a triplication wherein the internal copy is inverted (15). Inverted duplications have been identified as playing a role in adaptation and evolution (15,16) and have been associated with human pathologies and diseases (17,18). Recently Newman *et al.* (14) used Illumina sequencing on a human cohort of 112 people with CNVs and found ∼9% (11/119) of CNVs identified had inversions. As not all CNVs were able to be identified, this represents a conservative lower bound on their frequency. In our recent study of *de novo* CNVs in the yeast genome (16), ∼37% (14/38) of CNV breakpoints had inverted duplicated sequence, but at some loci as many as 89% of CNVs contained inverted duplicate sequence.

Here, we show that genomic regions containing inverted duplicated DNA sequence generate nanopore reads with pronounced base-calling errors, missed bases and low sequence quality (i.e. phred scores). We propose that this high error rate is caused by biophysical interference induced upon the translocating DNA by the formation of large-scale secondary structures involving the inverted duplicate sequences. We propose that the successive base-pairing induced by the formation of these structures acts as a ratchet to pull the translocating DNA through the nanopore at an increased rate thus increasing the current and decreasing the number of measurements made for each nucleotide. This decrease in sampling per nucleotide results in information loss and a decreased capacity of base-callers to differentiate between individual bases. This may present a technical hurdle that cannot easily be overcome with only improvements in machine learning models.

## MATERIALS AND METHODS

### Library preparation

All yeast strains were grown to greater than 1 × 10^7^ cells/mL in 30 mL minimal media (16) or YPD (Supplemental Table S1). Genomic DNA from each strain was extracted using Qiagen 20/G Genomic tips from ∼1.5 × 10^9^ cells using the manufacturer's protocol.

All genomic DNA was barcoded using Oxford-Nanopore's native barcoding genomic DNA kit (EXP-NBD104), adapters were added using the ligation sequencing kit (SQK-LSK109). The manufacturer's protocol (versions NBE_9065_v109_revB_23May2018 and NBE_9065_V109_revP_14Aug2019) was followed with the following exceptions: incubation times for enzymatic repair step were increased to 15 min. All Agencourt AMPure XP beads were incubated for 30 min at 37°C before elution. Adapter ligation time was increased to 10 min. Multiplexed libraries were loaded on MinION flowcells (FLO-MIN106D R9) and run on a MinION sequencer (MIN-101B).

### Base-calling and Alignment

Base-calling was performed using Albacore v2.3.4, Guppy v2.3.5, v3.0.3 and 3.1.5. Low-phred scores associated with junctions of inverted duplicated sequences were observed using each software. All subsequent analysis was performed using Guppy v3.1.5 with the ‘-fast5_out’ option enabled to save the associated squiggle data for each read. The raw electrical signal (e.g. squiggle) was extracted using SquiggleKit (19). Demultiplexing was performed using Epi2Me with default settings.

All alignments were made using minimap2 with the ‘-ax map-ont’ option (20).

### Low-phred score analysis and inverted duplicate junction identification

We developed a bioinformatics algorithm, Mugio, that uses fastq and bam files to visualize and quantify reads with low-phred scores and identify potential inverted duplicate sequence junctions.

Inverted duplicate junction identification is performed by first analyzing reads and their orientation to a reference genome. Defining a low-phred scoring region as a region at least 10 nucleotides long with a median phred score less than five standard deviations from the global median for the sample. On average, this results in 18% of all reads in a sample having at least one low-scoring region (Supplemental Table S2). Therefore, we further refine our criteria to limit our analysis to likely candidate inverted duplicate sequence junctions. First, because junctions represent an inversion, we require low-phred scoring reads map to the same chromosome in different orientations. Secondly, the boundaries of split-reads are used to identify the original location of the junction in the reference genome. We accomplish this by storing the boundaries of split-reads as pairs of reference genome coordinates. We use a greedy algorithm to resolve overlapping or conflicting boundary pairs. Finally, because we are specifically looking for duplications, we expect an increase in relative read-depth around the junction. Note, in this procedure we do not explicitly look for inverted duplicates of any size in the reference genome, or limit our analysis to exclude chromosomal features such as telomeres or centromeres.

This process is able to recapitulate 9 inverted duplicate junctions (A1–9, Supplemental Table S3) previously described by our lab using Illumina sequencing (16). Five additional inverted duplicate junctions were identified in these samples using mugio (B, C, T, in Supplemental Table S3). Because these are in low-complexity, repeat or Ty transposon regions they are intractable to identification using short-read sequencing. The rDNA locus was manually evaluated as the highly repetitive and highly duplicated region presents a difficulty for automated inverted duplication detection.

#### Determination of length of low-phred scoring regions

Reads containing a predicted inverted duplicate junction were scanned to identify all subregions (minimum 10 bases in length) that have a median phred score lower than five standard deviations from the global median. Subregions are expanded until the median phred-score in a 10 base window increases above the global median at which point the expansion of the subregion is halted and the subregion is stored for further analysis. Unbounded regions, which never recover from the low-phred score region before the read ends, were not included in length measurements. Pre-junction region lengths were measured from the beginning of the read to the beginning of the low-scoring region.

#### Determination of translocation rate

To determine if the translocation rate changes within a low-phred scoring region we first aligned the read to the reference genome to derive the expected number of nucleotides sequenced for each aligned and unaligned region (21,22) (see Supplemental Methods). We then divide the number of sensor sampling events for that region of the read by the number of expected bases. The estimated translocation rate is the number of samples per segment divided by the length of the corresponding segment in the reference genome.

## RESULTS

SVs and CNVs result in novel sequences that are often diagnostic of the mechanism of formation (3). Different classes of CNVs can result in inverted duplicated DNA sequence characterized by a nearly identical DNA sequence oriented in the opposite directions with a junction region separating them (Figure 1A). Some CNVs may contain multiple inverted duplicate sequences. We recently identified numerous CNVs containing ODIRA generated inverted duplications in experimentally evolved strains of *Saccharomyces cerevisiae* using short-read Illumina sequencing (16). ODIRA is a DNA replication-based mechanism of CNV formation (15,23) that generates CNVs with characteristic triplicated sequence wherein the internal repeat is inverted relative to the other two copies. Thus, these CNVs contain two inverted duplicate sequences. When the junction between the inverted sequences is unique it enables their identification using short-read sequencing. The structure of ODIRA breakpoints has been verified using orthogonal methods such as restriction digest analysis and Southern blotting (23). Although ODIRA CNVs motivated our original experiments, inverted duplications result from other CNV formation mechanisms as shown below.

We sought to use MinION long read sequencing to verify the structure of ODIRA CNVs. While these sequencing runs were largely successful (Supplemental Table S1 and Supplemental Figures S1 and S2), we were not able to identify the expected CNVs. Instead, we found that a majority of reads (80–100%) that span the predicted inverted duplicate junction exhibited low-phred scores and high-rates of mismatches with the reference genome (Supplemental Figure S3). Furthermore, these aberrant reads are clustered at the predicted junctions (Supplemental Figure S4). To ensure that sequencing failure at inverted duplicate junctions was not the product of library construction we verified that there was no substantial variance in sequencing quality (as defined by the phred score) between samples with and without inverted duplicate junctions (Supplemental Figure S5). Furthermore, we show that this is not a phenomenon specific to ODIRA as it was also observed for CNVs derived from tandem inverted duplications (Supplemental Table S5, Supplemental Figures S6–S8) Analysis of sequence reads that span inverted duplication junctions showed that the drop in sequencing quality occurred close to the junction sequence identified using short read sequencing (Figure 1C). In strains that do not contain inverted duplications there is no loss of sequence quality at the corresponding site and the sequence can be unambiguously determined (Supplemental Figure S10). We also compared sequence read performance to additional CNVs and SVs identified using Sniffles (24). We found no comparable failure in sequencing performance at loci identified as having insertions, deletions, or duplications (Supplemental Table S5). Notably, of the three inversions identified by Sniffles, a closer analysis found that two of these were actually inverted duplications, further emphasizing how sequencing failure impacts downstream analysis. Furthermore, we found evidence consistent with similar sequencing failure in human genomes sequenced using ONT’s PromethION platform (Supplemental Figure S9), suggesting that this phenomenon is not limited to either yeast genomes or the MinION platform.

We hypothesized that the low sequence quality at inverted duplicate junctions may reflect a change in the nanopore signal. An analysis of the raw current output from the sequencing pore revealed that the decrease in phred score corresponds with an ‘uplift’ in current (25) (Figure 1D, Supplemental Figure S11). We also sought to estimate the change in translocation rate across the different regions of the reads, as current is intimately connected with the translocation rate (21,22). We find that the uplifted regions also have a characteristic decrease in the number of samples per nucleotide consistent with an increased translocation rate of the DNA strand through the pore (Figure 1E, Supplemental Figure S12, see Materials and Methods section).

**Figure 2.**
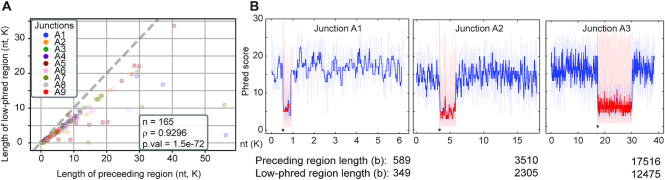
The length of the preceding sequenced region determines the length of the low phred scoring region. (**A**) For each Illumina sequenced, validated inverted duplicate junction (Junctions A1–9, Supplemental Table S3) we compared the length of the low-phred score region with the length of the preceding region that has a typical phred score for all independent sequence reads (see Materials and Methods). We find that the lengths of the high quality and subsequent low-quality region for each unique read are significantly positively correlated (Spearman rho = 0.93, *P*-value 1.5e–72). For all sequence reads, the preceding region is of slightly greater length than the low-phred scoring regions (the dashed line is the line of identity). This is consistent with the low-scoring region consisting of both miscalled bases and missed bases, potentially due to loss of resolution between bases. (**B**) Three archetypal reads of different sizes were selected from three different inverted duplicate junctions, despite differences in total read length the correlation in low-phred region length and preceding region length remains.

We find that the length of the aberrant uplifted regions and corresponding reduced phred score have a high degree of correlation with the length of sequence that precedes the inversion junction (Figure 2), which is otherwise of typical quality (see Materials and Methods section). This high degree of correlation between the lengths of high quality and low-quality bases within a single read was only observed for structural variants that have inverted duplicated sequence (Supplemental Tables S3 and S4). Although highly correlated, the lengths of the reduced phred score regions were consistently shorter than that observed for the preceding region, suggesting that these regions not only featured inaccurately called bases but missed bases as well. This decreased accuracy in base-calls and the increased frequency of missed bases is consistent with a loss of information caused by the increased translocation rate and concomitant decrease in sampling rate per nucleotide.

Furthermore, we assessed a published RNA sequencing data (26) set for sequence reads that are diagnostic of sequence failure associated with inverted duplicate sequences. We did not detect any evidence that this occurs in RNA sequencing data, which may be a result of the greatly reduced read length (average median read length of 952 nucleotides) or the relative paucity of inverted duplicated sequences in the transcriptome.

We attempted to use a variety of base callers on reads with uplifted regions, but found that no base caller can accurately perform base-calling within these regions. Because nanopore sequencing relies on a neural network algorithm to perform base-calling (e.g. (10)), the accuracy of base-calling is dependent on how similar the input is to the training data. Not only does the current in the uplifted region exceed the range of signals used to train the base caller, but the uplifted region also has a decreased sampling frequency, decreasing the total information available to the base-caller. As such, existing base-calling algorithms are unable to perform well, resulting in miscalls, skipped bases, and low phred scores. While descriptions of uplifted signal have been previously observed by some members of the Nanopore Community these were largely anecdotal and in relation to the now discontinued 1D2 technology. To our knowledge, sequencing failure associated with self-complementarity has not been reported for biological sequences. Furthermore, because the Nanopore Community is located behind a paywall this knowledge has heretofore been unavailable to the broader scientific community.

## DISCUSSION

We have identified a phenomenon in which inverted duplicate sequences result in a substantial and significant decrease in the sequence quality of nanopore long reads, confounding the ability to define these sequences in CNVs and SVs using nanopore sequencing. We show that the loss of sequence quality correlates with increased translocation rates that generate a systematic uplift in the detected current. This increased translocation rate also decreases the sampling rate leading to inaccurate base-calls and missed bases. Finally, we find the length of this low phred-score, uplifted, region is a function of the length of the preceding complementary sequence that is of otherwise normal quality.

**Figure 3.**
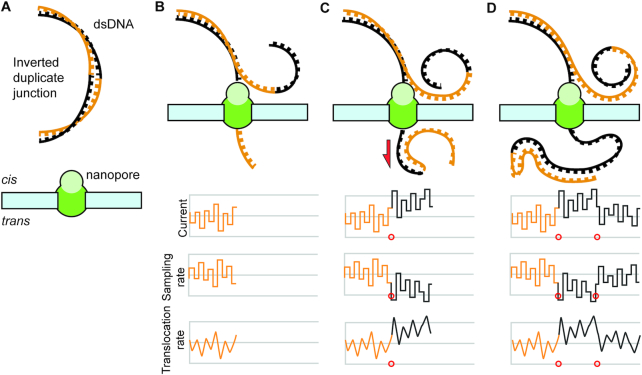
Proposed model for DNA secondary structure interference with sequencing pore. Double stranded DNA containing an inverted duplication (orange and black strands) is introduced to a sequencing pore (**A**). As the strand translocates through the pore, sensors sample the change in the current, and record the measurement (**B**). Once the junction has passed over to the *trans* side of the nanopore, the likelihood for the inverted duplication to form a secondary structure increases dramatically (**C**). As these secondary structures form (first red circle), they increase the pull on the translocating strand (red arrow), thus increasing the translocation rate, causing the sampling rate to decrease and the current to increase. Once the translocating strand is past the inverted duplication region the interference of the secondary structure decreases (second red circle) with a concurrent relaxation of the translocation rate, an increase in sampling rate, and a decrease in current (**D**).

Because current is a property of translocation rate (27) we propose that the uplift in current is consistent with altered rates of DNA translocation through the pore. Translocation rates could be altered by the intramolecular base-pairing of the complementary sequences on either side of the junction acting as ratchet-like mechanism on the DNA strand or as physical interference generated by interactions between the secondary structures and the nanopore (Figure 3). This phenomenon is similar to the observed current uplift in ONT’s now discontinued 2D chemistry which used small oligo linkers to join double-stranded DNA into large self-complementary single strands (25). Further support for this model is the proximity of the inverted duplicate junction to the low-phred scoring regions; and the high correlation between the region lengths before and after the junction.

Our biophysical model for this aberrant behavior suggests that the frequency and magnitude of sequencing failures will be dependent on parameters that drive secondary structure formation, such as temperature, salt concentrations, fragment size, and the capacity of any given sequence to self-anneal, all of which will make the generation of a generalizable machine learning model difficult. Notably, because inverted duplications lack specific sequences, solutions developed that target specific sequences (25) would be infeasible. Additionally, while the introduction of nucleases on the trans-side of the membrane (25) would mitigate the formation of large scale secondary structures, the active degradation of the sequenced strand may induce biophysical forces on that strand that could also alter the translocation rate. One possible strategy would be to use a biophysical or chemical approach to decrease the rate of secondary structure formation, for example, by using asymmetric salt concentrations to decrease the free-energy of base-pairing (28).

Finally, we note that we identified this aberrant behavior by virtue of targeted analysis of predicted inverted duplicated sequence. As most analysis tools exclude low quality base calls, we expect that inverted duplicated sequences in genomes of unknown structure may be missed using current approaches.

## DATA AVAILABILITY

Sequence files (Fastq) for all strains referenced in this work are available online from NCBI SRA under the accession PRJNA591579: https://www.ncbi.nlm.nih.gov/bioproject/PRJNA591579.

All computer code is available in the Github repository: https://github.com/GreshamLab/mugio.

## Supplementary Material

gkaa206_Supplemental_Files
