# Supplementary material for: Inverted duplicate DNA sequences increase translocation rates through sequencing nanopores resulting in reduced base calling accuracy
Source: Nucleic Acids Res. 2020 Apr 7;48(9):4940–5. doi: 10.1093/nar/gkaa206 (PMC7229812; doi:10.1093/nar/gkaa206)
Supplement: gkaa206_Supplemental_Files [file gkaa206_supplemental_files.zip › Supplementary_Material.pdf]

**Supplementary Tables:**

**Supplementary Table 1: Strains used in this study.**
Table includes our internal strain reference ID, SRA Accession number, brief description, and growth media. Also includes Mean Sequencing Depth for the nuclear genome and each chromosome.

**Supplementary Table 2: Low-phred score regions.**
For each strain certain properties of low-phred scoring regions are supplied such as the Lower Threshold (lt), Middle Threshold (mt), Upper Threshold (ut), and Standard Deviation of the distribution of phred-scores from the data. These terms also define to parameters used in inverted duplication junction detection performed by mugio (Supplemental Methods section). The results of that analysis are listed in the subsequent columns; Pct of reads with low phred score regions, Median number of regions per read, Median region length (nt), Minimum length, Maximum Length, Median median low phred score, Minimum median low phred score, Median Maximum phred score.

**Supplemental Table 3: Structural Variant information.**
This table summarizes structural variants included in our analysis of sequencing failure. Each structural variant is given a categorical ID in the Structural Variant Breakpoint ID column (also used in Supplemental Tables 4 and 5), as well as the strain ID. A brief description is provided under SV Description. Loci identified as having a SV breakpoint by Illumina short-read sequencing are listed under the Short Read Detected Structural Variant Breakpoint column. Using mugio and ONT long-read data correlations of Pre-junction phred-scores and post-junction phred scores are reported under the Length Correlation column. The last three columns describe if the SV was identified as an inverted duplication (ID) using either Illumina (Validated as ID using Illumina (Lauer et al. 2018)) or ONT long-reads using mugio (Identified as ID by Mugio) or Sniffles (Identified as ID by Sniffles).

**Supplemental Table 4: Results of Mugio analysis**
This table features the candidate inverted duplicate junctions (Candidate Locus) resulting from mugio ran in detection mode (-bprd). When these loci overlap with breakpoints previously evaluated (Supplementary Table 3) the Structural Variant Breakpoint ID is given. These Candidate loci were also evaluated by mugio ran in evaluation mode (-e) with the results listed in columns Length Correlation (Rho), Rho, pval, Supporting reads and Total reads. Supporting reads are reads with significant low phred-scoring regions bounded on both sides by normal phred-scoring regions. Any candidate with at least 5 supporting reads and a *Rho* correlation greater than 0.5 and a p-value less than 0.2 is assumed to be an inverted duplication.

**Supplemental Table 5: Results of Sniffles analysis**
This table features all significant SVs (Candidate Locus) identified by Sniffles as well as the predicted SV. These loci were also evaluated using mugio as described above.

## Supplementary Figures

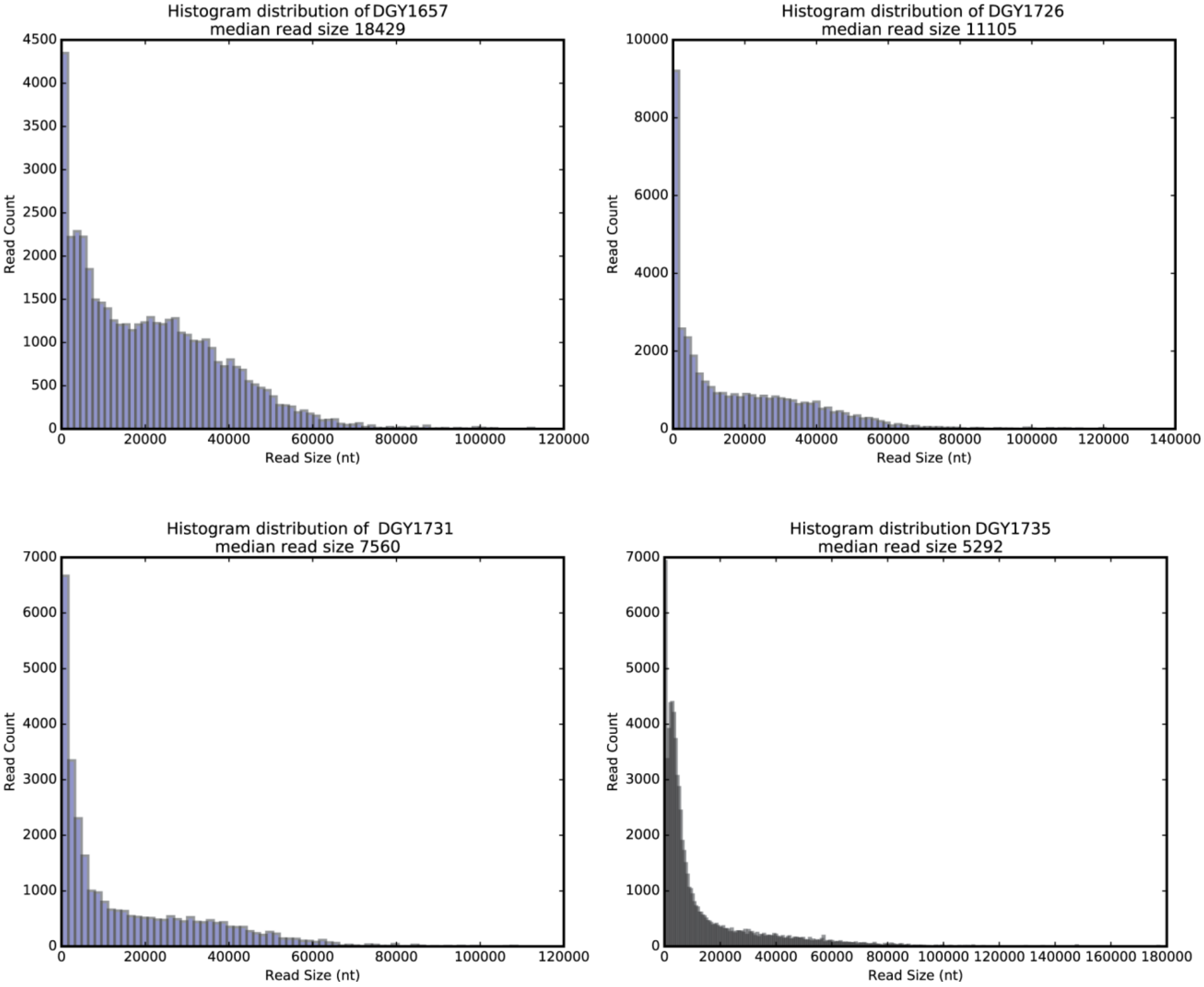


**Supplemental Figure 1. Histogram Distributions of Samples DGY1657, DGY1726, DGY1731, and DGY1735.** Distributions calculated from number of called bases per read in the respective FASTQ after demultiplexing. Median read size calculated without removal of outliers. For a list of all strains used in this study please refer to Supplemental Table 1.

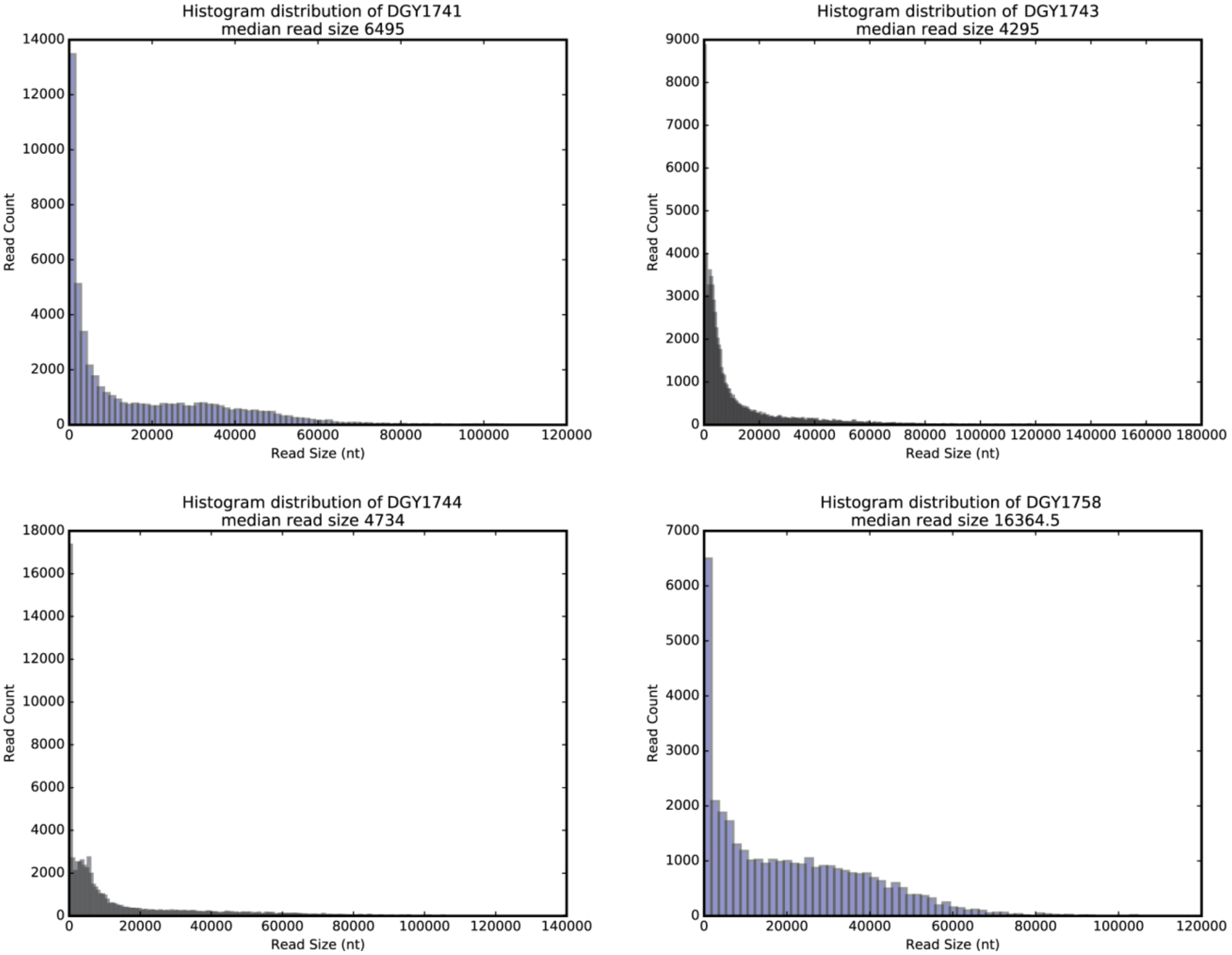


**Supplemental Figure 2. Histogram Distributions of Samples DGY1741, DGY1743, DGY1744, and DGY1758.** Distributions calculated from number of called bases per read in the respective FASTQ after demultiplexing. Median read size calculated without removal of outliers. For a list of all strains used in this study please refer to Supplemental Table 1.

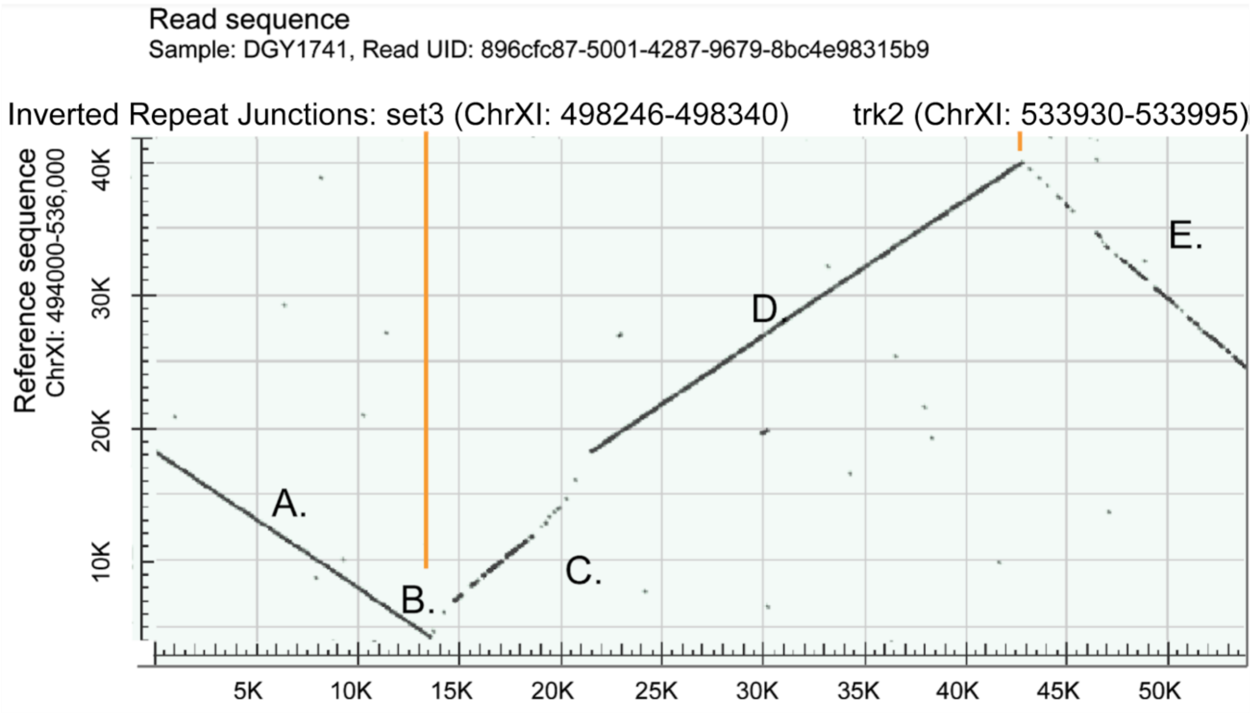


**Supplemental Figure 3. Dot-plot comparison of a read spanning two predicted inverted duplicate junctions relative to the reference sequence.** The horizontal axis represents the sequence of a single long read (UID: 89ccfc87-5001-4287-9679-8bc4e98315b9) that spans two inverted duplicate junctions (orange lines). This is the same read shown in **Figure 1**. The reference sequence is from ChrXI: 494,000 - 536,000. Significant sequence matches between the two are represented by a black dot. The initial solid down-sloping segment represents a close alignment between the read and the negative strand of the reference (**A**). At the inverted duplicate junction, the direction of the slope changes denoting the inversion (**B**). Note that the length corresponding to the preceding sequence is poorly aligned and of shorter length than the reference (**C**). Once the sequence is past the duplicated region that is contained within the DNA molecule the alignment improves (**D**) until it encounters the second inverted duplicate junction when the alignment again degrades (**E**). The read ends before the sequence again recovers.

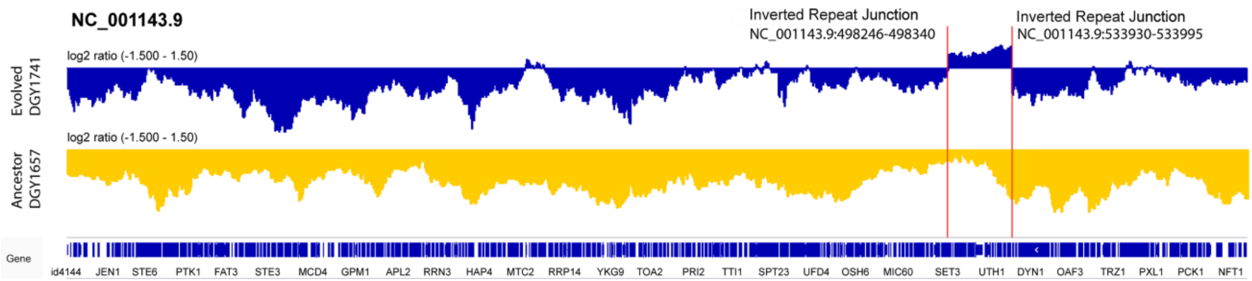


**Supplemental Figure 4**. **Clustering of reads containing a contiguous span of significant low-phred scoring regions.** The distribution of reads with significant low-phred scoring regions (see Methods) at the same locus in two yeast strains. For each strain we calculated the log2 transformed ratio of low-phred scoring reads (reads with a minimum of length of 50 contiguous nucleotides with a phred score greater than 3 standard deviations from the global median) to total reads, using Deeptools (1). The bottom track shows the read distribution for the wildtype strain which lacks inverted duplicate junctions. The top track shows the read distribution for a strain that has inverted duplicate junctions (red lines). The pronounced peak of reads with significant low-phred scoring regions clustered around the two junctions in the evolved strain this is entirely absent in the wildtype strain.

1. Ramírez, Fidel, Devon P. Ryan, Björn Grüning, Vivek Bhardwaj, Fabian Kilpert, Andreas S. Richter, Steffen Heyne, Friederike Dündar, and Thomas Manke. deepTools2: A next Generation Web Server for Deep-Sequencing Data Analysis. Nucleic Acids Research (2016). doi:10.1093/nar/gkw257.

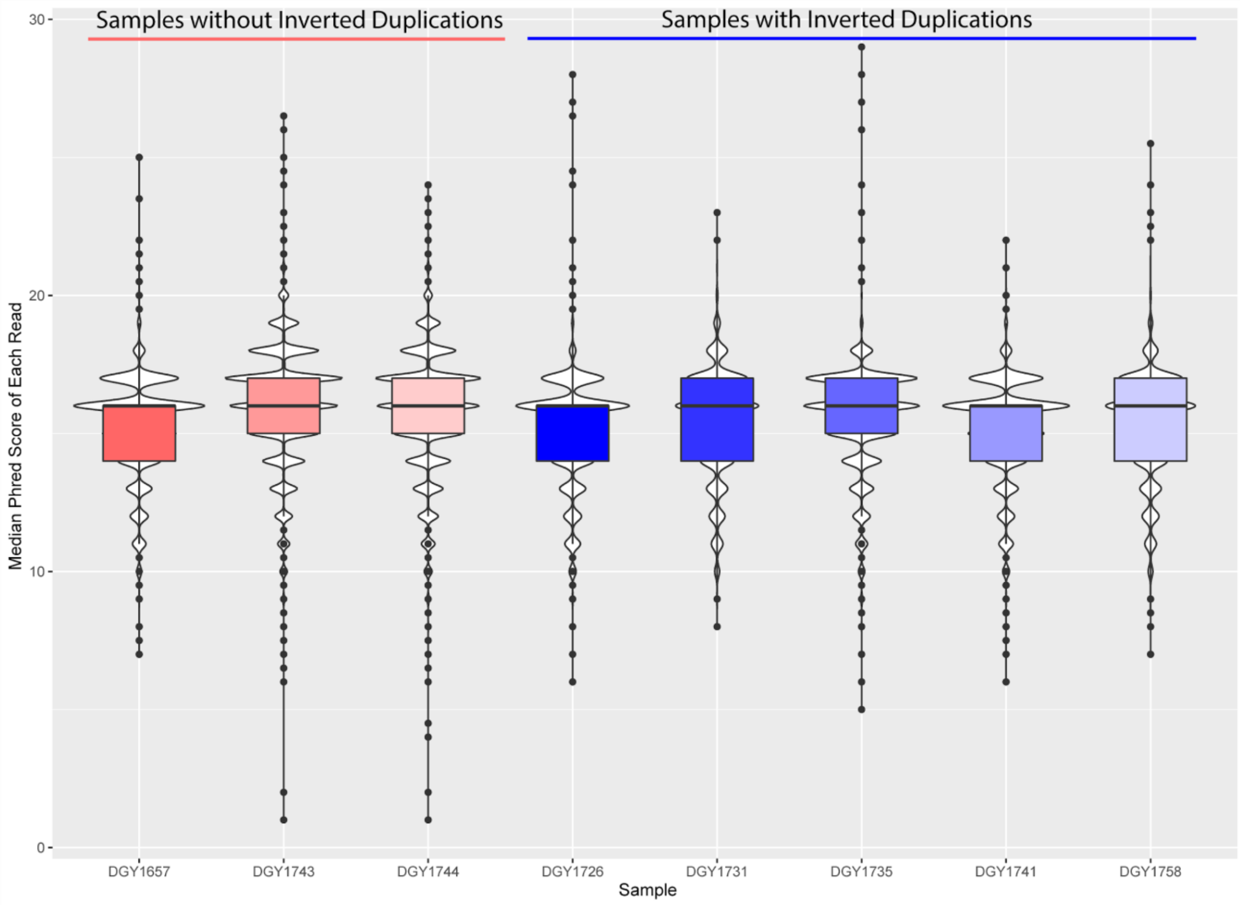


**Supplemental Figure 5. Sequence quality is not globally compromised in genomes containing inverted duplicate sequences.** The distribution of phred scores of all reads for each sample. The mean of every distribution is within 1.05-fold of the mean of a wildtype strain that does not contain any SVs. There is no substantial difference between the samples without predicted inverted duplicate (red) and those with (blue).

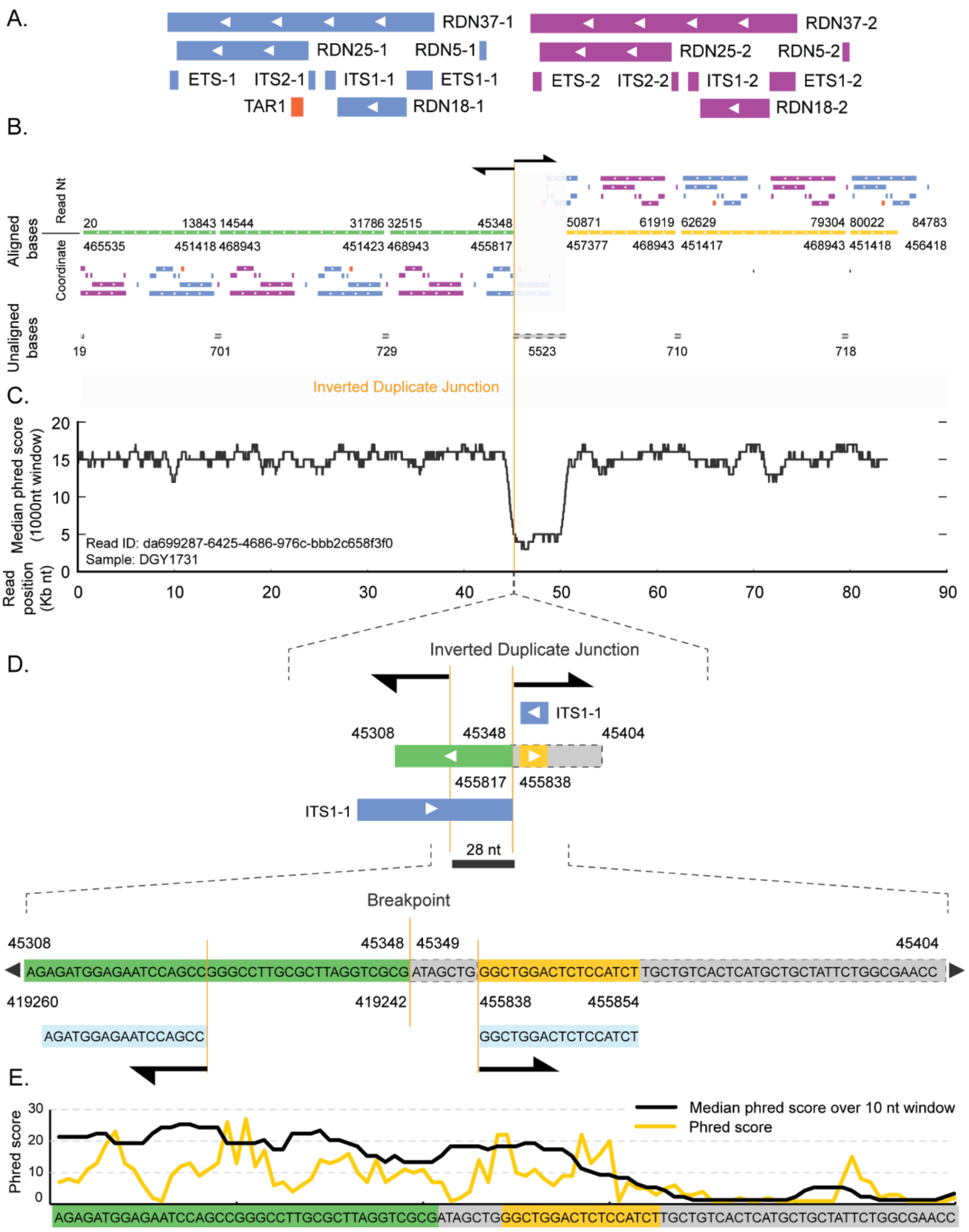


**Supplemental Figure 6. An inverted duplicate junction at the yeast rDNA locus.** A schematic of the genetic architecture of the rDNA locus (A). The hypothetical structure that

generated the read with the orientation of each segment is relative to the reference genome shown (forward in yellow, reverse in green) (B). A median phred-score trace showing the characteristic decrease in phred-score at the inverse duplication junction (C). A schematic of the junction showing the orientation of the aligned and unaligned segments, with the complementary sequences that form the secondary structure subset and highlighted in blue (D). An analysis of the phred-score shows that a sustained decrease occurs after the complementary sequence (E).

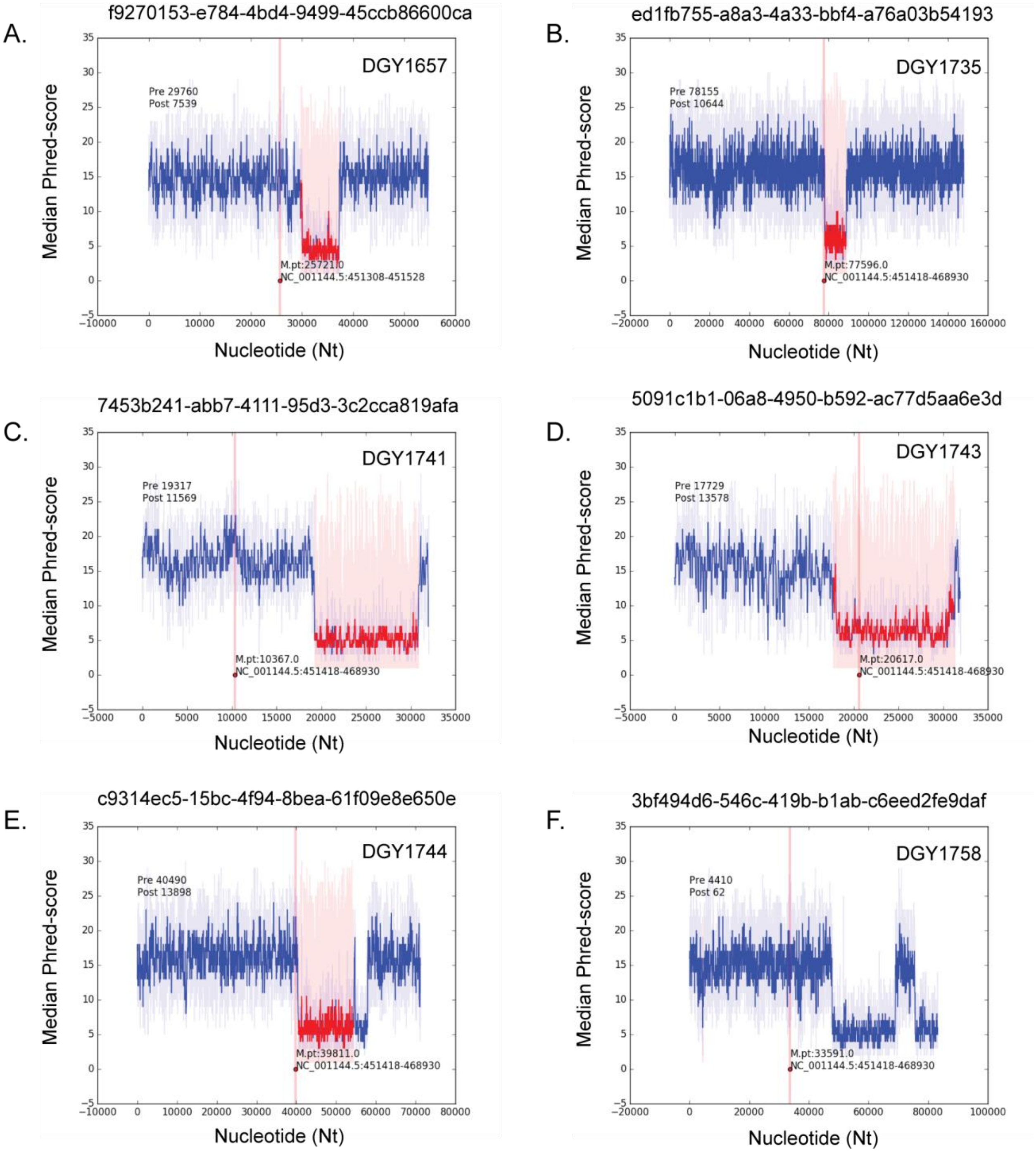


**Supplemental Figure 7. Prevalence of sequencing failures at rDNA locus across multiple samples.** Here we show reads from every sample (barring DGY1731, which is shown in Supplemental Figure 8). These reads feature low-phred scoring regions and have alignments that support inversion with duplication (A-F). These reads were manually curated as our inverted duplication identification pipeline does not work well for the highly duplicated rDNA locus.

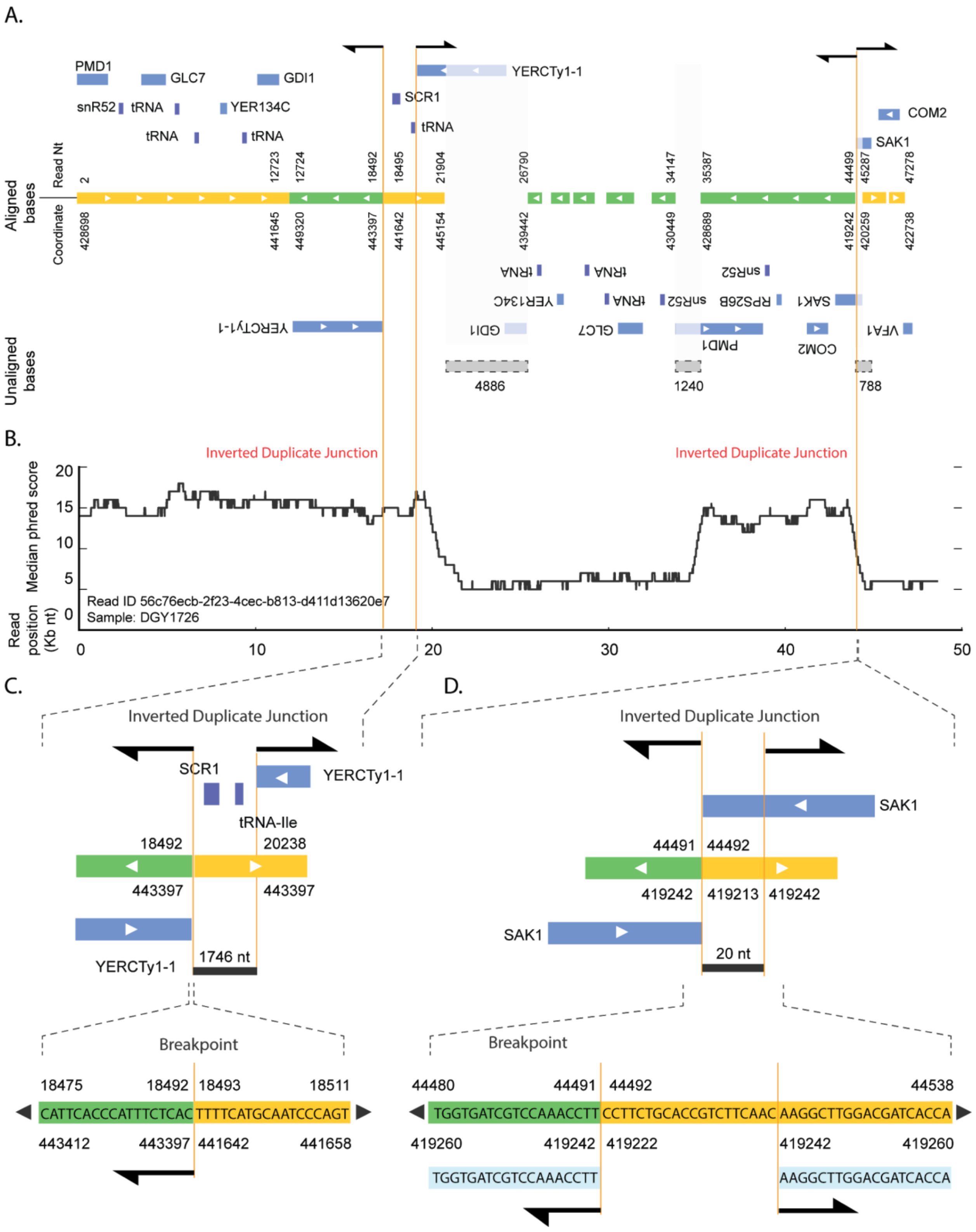


**Supplemental Figure 8. Two inverted duplicate junctions formed by two different mechanisms.** The hypothetical structure that generated the read with the orientation of each

segment is relative to the reference genome shown (forward in yellow, reverse in green) (A). A median phred-score trace (B). A schematic of the junction showing the orientation of the aligned and unaligned segments for the YERCTy1-1 junction (C) and the SAK1 junction (D). Notably, for the SAK1 junction the complementary sequences that form the secondary structure subset are highlighted in blue. This is not shown for the YERCTy1-1 junction as this locus has a 1746nt length between the complementary sequences. Importantly, the phred-score dynamics at the two sites are different. The SAK1 site shows a decrease in median phred score starting very close (at Read nt 44499) to the breakpoint (at Read nt 44491). While the YERCTy1-1 site shows a larger distance between the decrease in median phred score (Read nt 21904) and the breakpoint (Read nt 18492). The increase in distance observed at the YERCTy1-1 site could be reflective of the decreased likelihood of interaction between the two complementary regions, suggesting that the proximity of complementary sequences can be a driver of the rate of secondary structure formation.

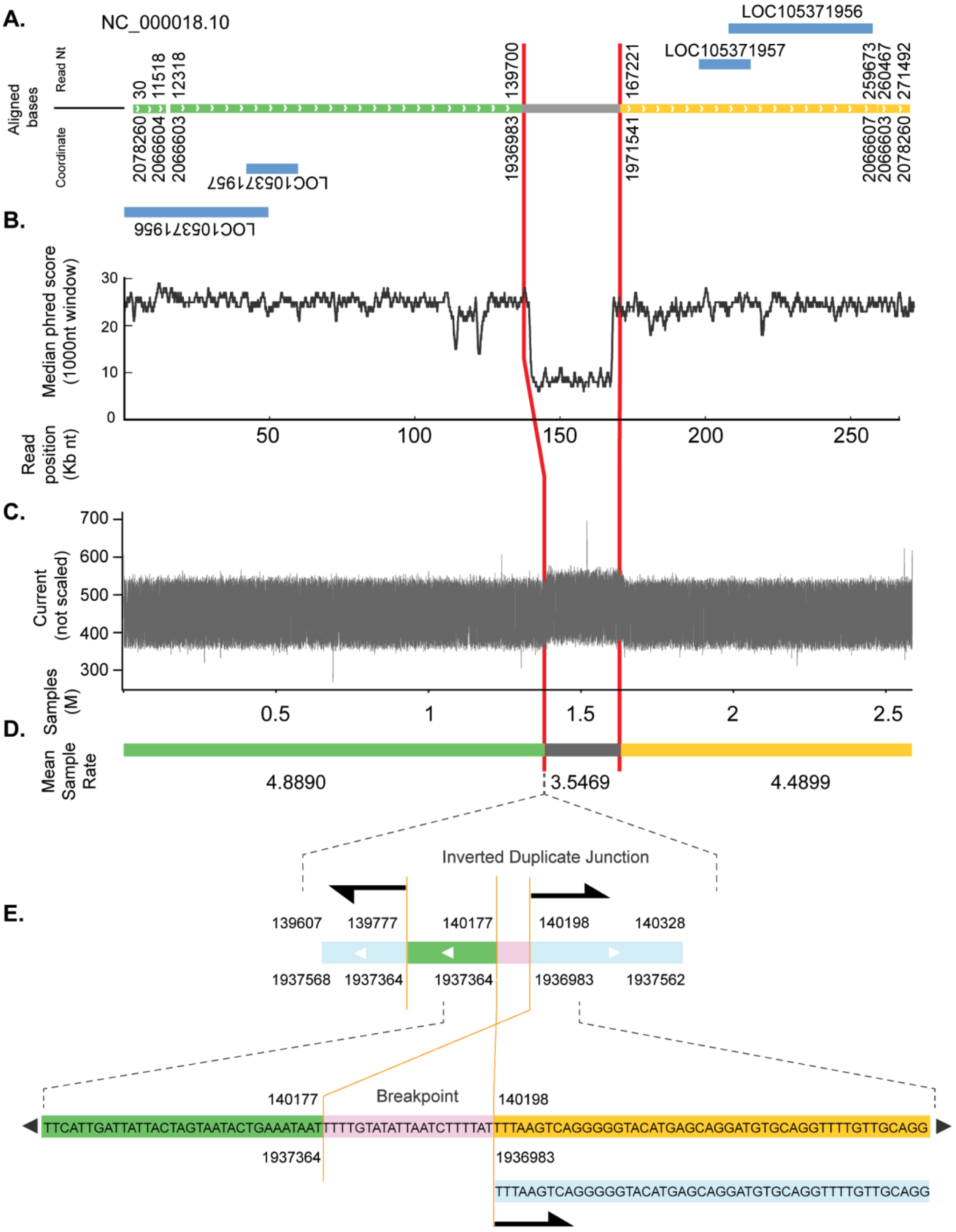
A.
NC_000018.10
LOC105371956
LOC105371957
Aligned bases
Read Nt
Coordinate
30
11518
12318
139700
167221
259673
260467
271492
2078260
2066604
2066603
1936983
1971541
2066607
2066603
2078260
LOC105371957
B.
LOC105371956
Median phred score (1000nt window)
Read position (Kb nt)
C.
Current (not scaled)
Samples (M)
D.
Mean Sample Rate
4.8890
3.5469
4.4899
Inverted Duplicate Junction
E.
139607
139777
140177
140198
140328
1937568
1937364
1937364
1936983
1937562
Breakpoint
140177
140198
TTCATTGATTATTACTAGTAATACTGAAATAATTTTTGTATATTAATCTTTTATTTTAAGTCAGGGGGTACATGAGCAGGATGTGCAGGTTTTGTTGCAGG
1937364
1936983
TTTAAGTCAGGGGGTACATGAGCAGGATGTGCAGGTTTTGTTGCAGG

**Supplemental Figure 9. Inverted duplication identified using human DNA.** The hypothetical structure that generated the read with the orientation of each segment is relative to the reference genome shown (forward in yellow, reverse in green) (**A**). A median phred-score trace showing the characteristic decrease in phred-score at the inverse duplication junction (**B**). The unscaled current measured per sensor sample (**C**). The mean sample rate per read segment, wherein the number of samples is divided by the number of bases the read spans in the reference genome (**D**, see Supplemental Methods). A schematic of the junction showing the orientation of the aligned and unaligned segments, with the complementary sequences that form the secondary structure subset and highlighted in blue (**E**). Note that the 'Breakpoint' sequence (pink) occurs in opposite orientations at two nearby loci in the genome (NC_000018.10:1936962-1936983 and NC_000018:1937364-1937385), yet this sequence only occurs once in the read but spans the two opposite orientations. This is consistent with a microhomology mediated CNV.

Sequencing data from CliveOME, version 3, performed using PromethION platform (2).

2. Brown, C. (2019). Cliveome ONT-HG1. [online] Available at: https://github.com/nanoporetech/ONT-HG1 [Accessed 27 Jan. 2020].

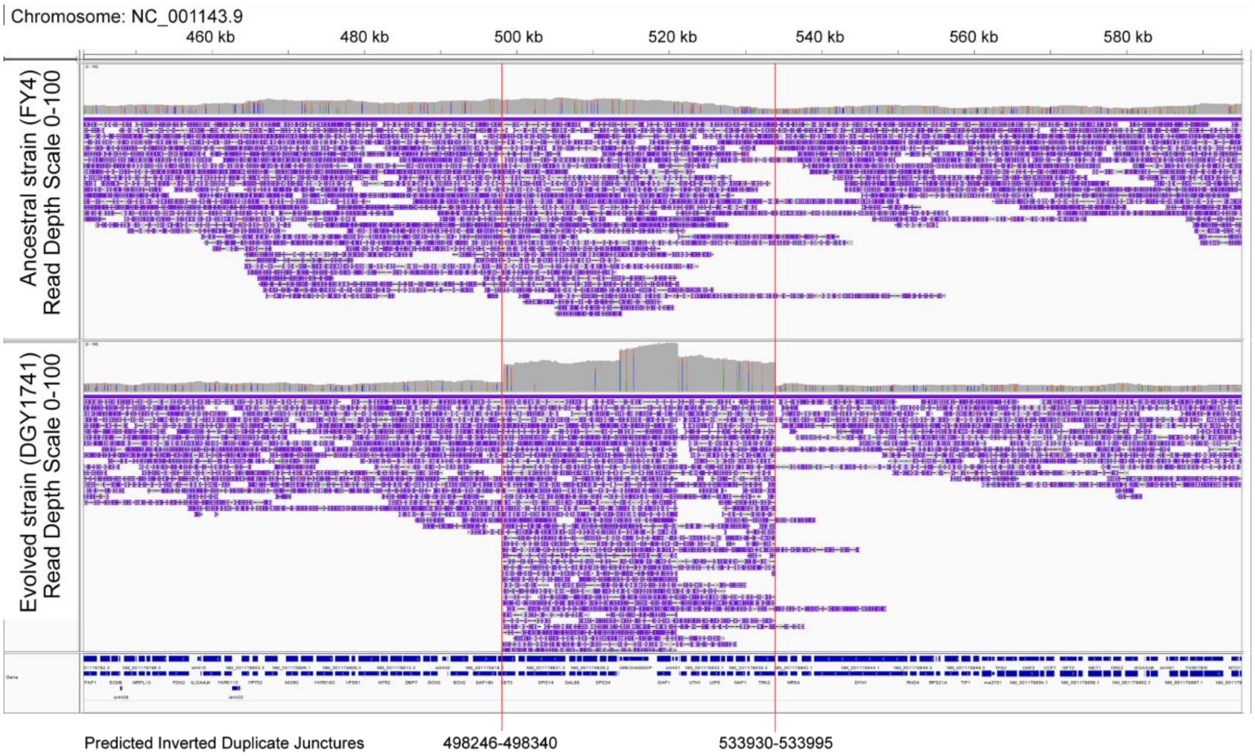


**Supplemental Figure 10**. **Frequency of low-phred scoring reads at a site with inverted duplicate junctions.** A comparison of aligned reads with significant low-phred scoring regions (reads with a minimum of length of 50 contiguous nucleotides with a phred score greater than 3 standard deviations from the global median) between the wildtype strain (top track) and a strain with predicted inverted duplicate junctions (bottom track). Note the substantially higher abundance of low-scoring reads in the strain with predicted inverted duplicate junctions.

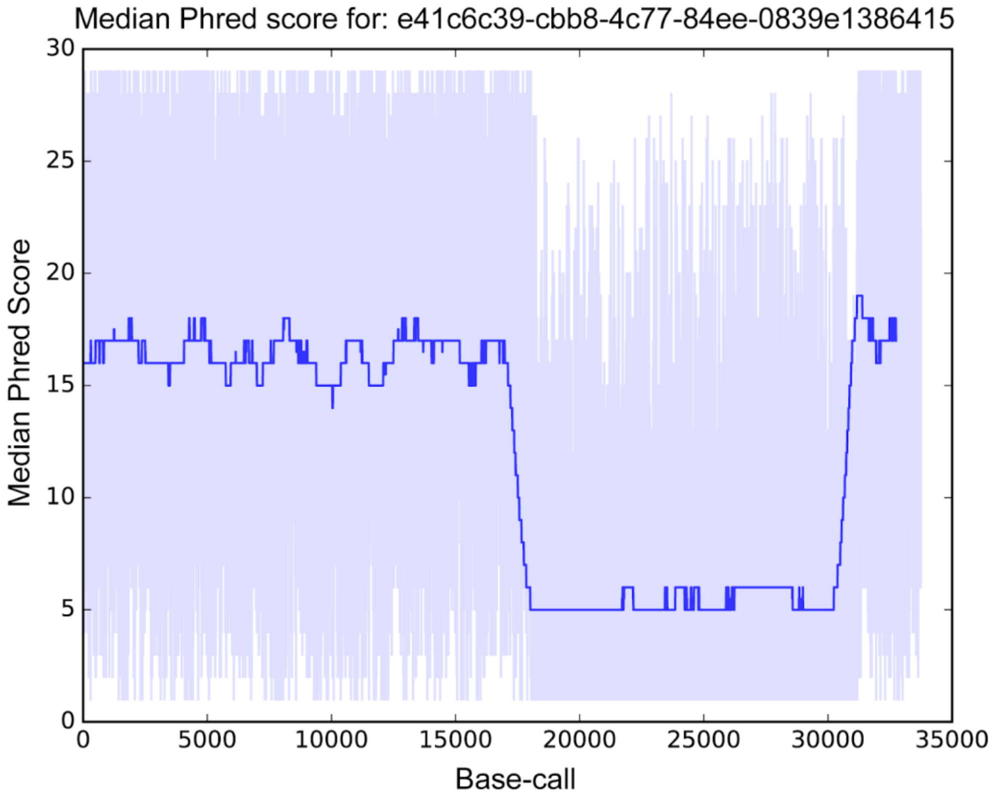
Median Phred score for: e41c6c39-cbb8-4c77-84ee-0839e1386415
Median Phred Score
Base-call
0
5
10
15
20
25
30
0
5000
10000
15000
20000
25000
30000
35000

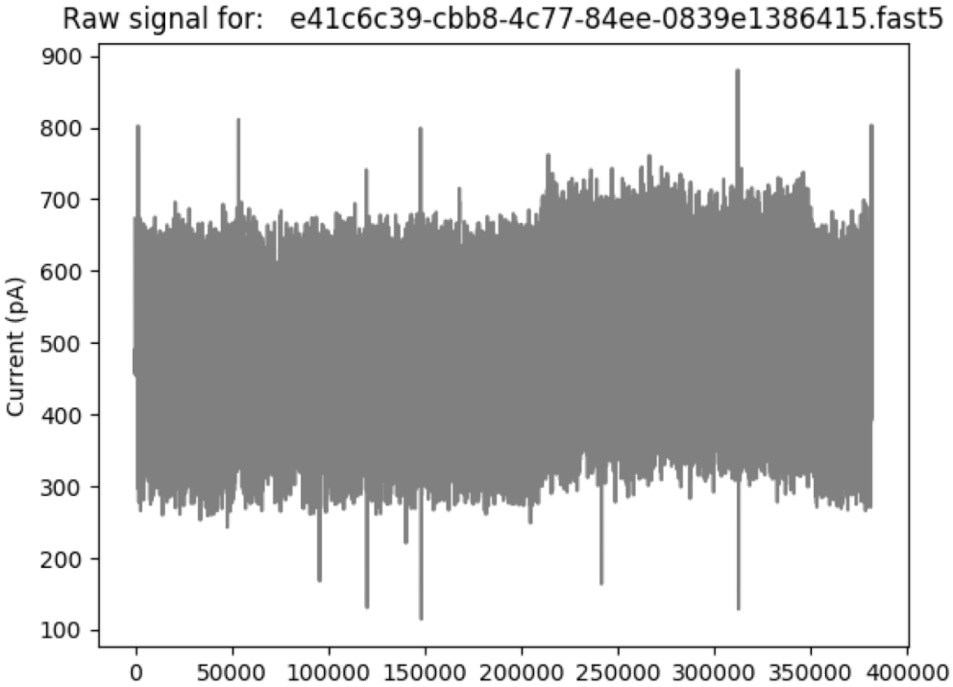
Raw signal for: e41c6c39-cbb8-4c77-84ee-0839e1386415.fast5
Current (pA)
100
200
300
400
500
600
700
800
900
0
50000
100000
150000
200000
250000
300000
350000
400000

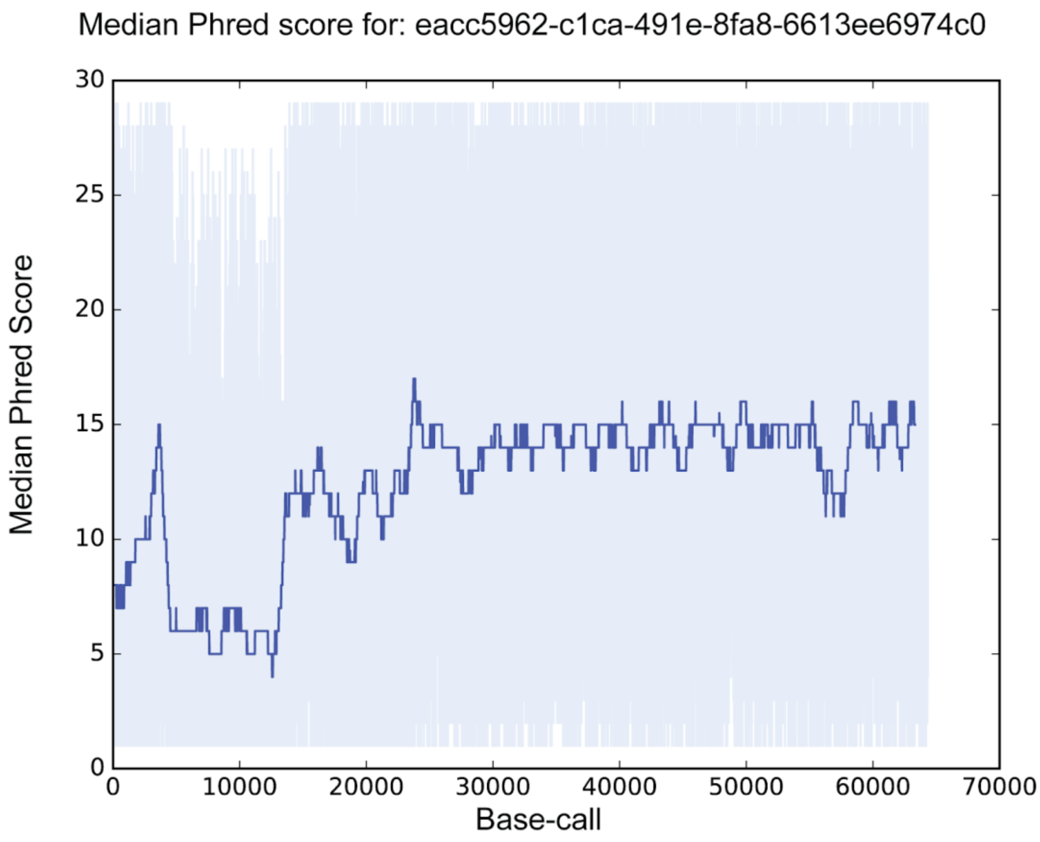
Median Phred score for: eacc5962-c1ca-491e-8fa8-6613ee6974c0
Median Phred Score
Base-call
0
5
10
15
20
25
30
0
10000
20000
30000
40000
50000
60000
70000

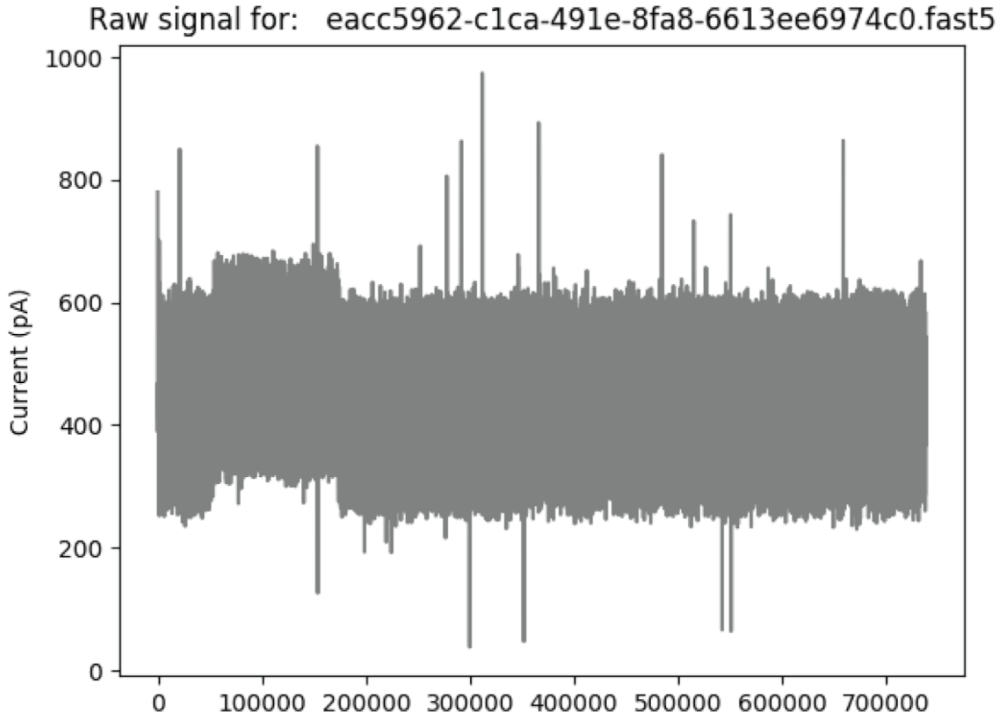
Raw signal for: eacc5962-c1ca-491e-8fa8-6613ee6974c0.fast5
Current (pA)
0
200
400
600
800
1000
0
100000
200000
300000
400000
500000
600000
700000

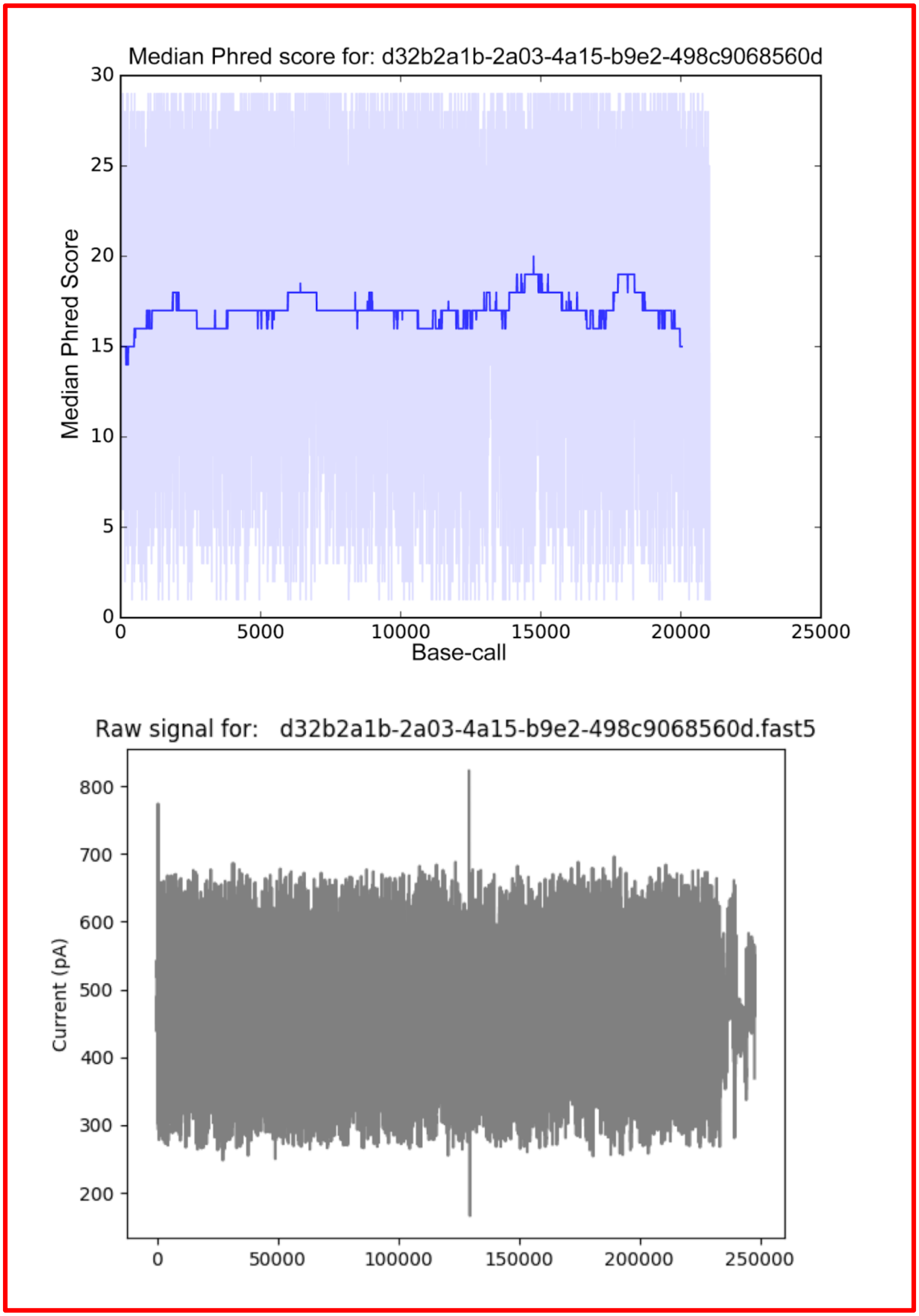


**Supplemental Figure 11**. Additional paired windowed phred-score and nanopore raw squiggle results. The first two examples have inverted duplicate junctions, resulting in coincident decreases in phred score and an aberrant squiggle readout. The third example (red border) is a read from a region without an inverted duplicate junction that lacks both of these properties.

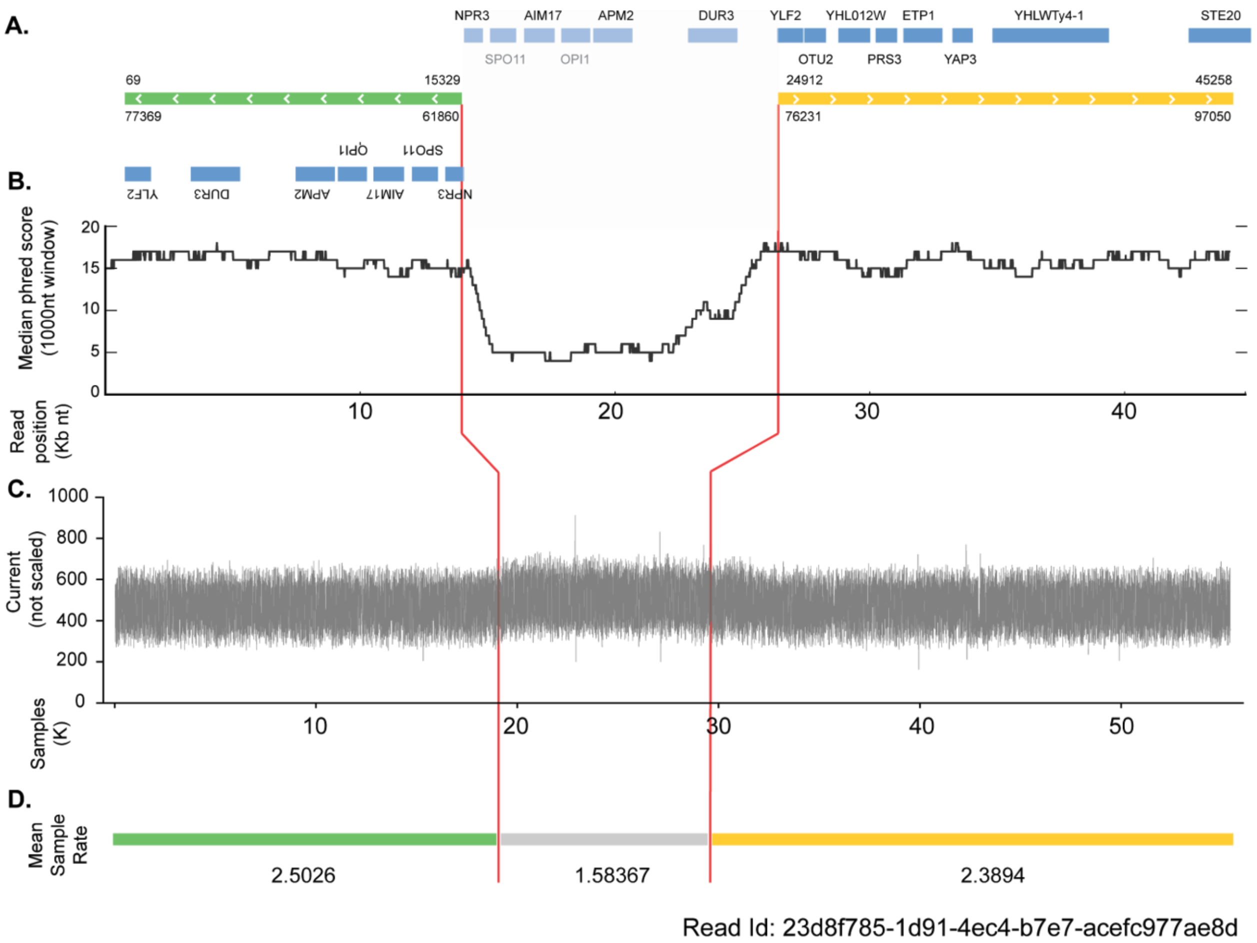


**Supplemental Figure 12. Low-phred scoring region exhibits decreased translocation rate of DNA through Nanopore.** The hypothetical structure of the read with the orientation of aligned segments to the reference genome indicated by color (with yellow as forward and green as reversed) (**A**). The median phred-score of the read calculated over a 1000nt rolling window (**B**). The unscaled current measured per sensor sample (**C**). The mean sample rate per read segment, wherein the number of samples is divided by the number of bases the read spans in the reference genome (**D,** see Supplemental Methods).

**Supplemental Methods:**

**Detection of Inverted Duplicate Junctions**

Although Illumina sequencing can be used to identify some inverted duplication breakpoints, as described previously (3), short-read sequencing is unable to reliably resolve breakpoints within low-complexity or repeat regions as these regions do not generate a unique sequence.

Attempts to identify breakpoints using long-read sequencing from ONT have to account for the low-phred score regions and their lack of alignment to the reference genome. Our tool Mugio allows for the identification of candidate inverted duplication breakpoints in ONT data sets by finding coherent clusters of significantly sized low-phred score regions (Supplemental Methods Figure 1).

Mugio first imports both the ONT fastq and a minimap2 generated sam file. Alignments in the sam file are removed if they are “multimappers” (SAM flag “not_primary_alignment” equals 1) or are unaligned to the reference genome (**Step 1**). Furthermore, reads are required to be “split reads”, that is at least on alignment with a SAM Flag “supplementary_alignment” equal to 1. (**Step 2**).

Reads that meet these criteria are then scanned for Soft or Hard clipping (‘C’ or ‘H’ in CIGAR) regions in excess of 100nt in length (**Step 3**). If such a region is detected the most proximal coordinate with an aligned region (‘M’ in Cigar) is assumed to be the most reliable candidate breakpoint. This coordinate is saved in the ‘starmap’ data object used at a later step.

Reads are further filtered to identify those reads that have over 1000 nucleotides that are significantly low-phred scoring and wherein those reads form at least one run of 100nt in length or greater (**Step 4**). Here a significantly low-phred score is defined as a phred-score less than 5 standard deviations from the median.

We next generate ‘anchorpoints’ to define the boundaries of the breakpoints (**Step 5**). We record the coordinates of the endpoints of the aligned segments of each read, these will necessarily include the most proximal matching coordinate of the low-phred region. We call these ‘anchorpoints’ as they act to anchor the unalignable sequence of the low-phred scoring regions. Once anchorpoints are generated, they are combined if close or overlapping and filtered if having a low score or ‘weight’. A weight is calculated for all anchorpoints, this is the sum of all reads over the length of the anchorpoints with an additional weight added if the anchorpoints span a Soft/Hard Clipped point in the Starmap. Each anchorpoint must have a minimum median read depth (default of 5) or be removed from further consideration.

Finally, we also require each anchorpoint to be within a read depth transition region (**Step 6**). These regions are defined as two halves - a 1000nt upstream of the anchorpoint and another 1000nt downstream. The median read-depth between these regions must be significantly

different (greater than 1.25-fold difference with a p-value < 0.05, Binomial exact test) with a minimum of 20 reads combined.

Any anchorpoint matching these criteria is then graduated to Inverted Duplication Breakpoint candidate status. These anchorpoints are reported in BED file format with their final score (**Step 7**). As the pipeline has many parameters that are derived from the data or set by the user the accuracy and precision of these candidates' breakpoint should be considered tentative until further investigated using the evaluation command.

3. Lauer S, Avecilla G, Spealman P, Sethia G, Brandt N, Levy SF, et al. Single-cell copy number variant detection reveals the dynamics and diversity of adaptation. PLoS Biol. 2018 Dec;16(12):e3000069.

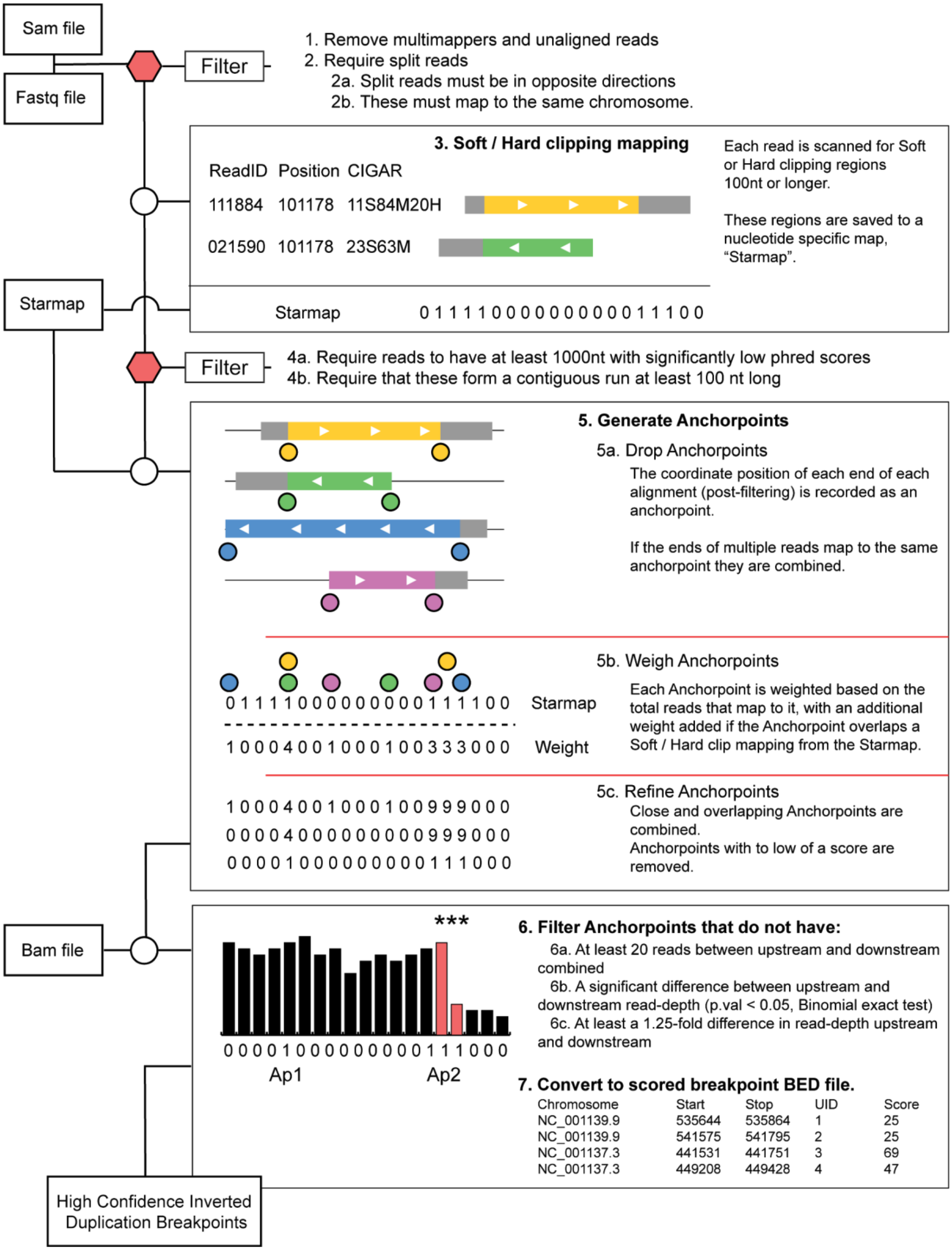
Sam file
Fastq file
Filter
1. Remove multimappers and unaligned reads
2. Require split reads
2a. Split reads must be in opposite directions
2b. These must map to the same chromosome.
3. Soft / Hard clipping mapping
ReadID Position CIGAR
111884 101178 11S84M20H
021590 101178 23S63M
Each read is scanned for Soft or Hard clipping regions 100nt or longer.
These regions are saved to a nucleotide specific map, “Starmap”.
Starmap
Starmap 0 1 1 1 1 0 0 0 0 0 0 0 0 0 0 1 1 1 0 0
Filter
4a. Require reads to have at least 1000nt with significantly low phred scores
4b. Require that these form a contiguous run at least 100 nt long
5. Generate Anchorpoints
5a. Drop Anchorpoints
The coordinate position of each end of each alignment (post-filtering) is recorded as an anchorpoint.
If the ends of multiple reads map to the same anchorpoint they are combined.
5b. Weigh Anchorpoints
0 1 1 1 1 0 0 0 0 0 0 0 0 0 0 1 1 1 0 0 Starmap
1 0 0 0 4 0 0 1 0 0 0 1 0 0 3 3 3 0 0 0 Weight
Each Anchorpoint is weighted based on the total reads that map to it, with an additional weight added if the Anchorpoint overlaps a Soft / Hard clip mapping from the Starmap.
5c. Refine Anchorpoints
1 0 0 0 4 0 0 1 0 0 0 1 0 0 9 9 9 0 0 0
0 0 0 0 4 0 0 0 0 0 0 0 0 0 9 9 9 0 0 0
0 0 0 0 1 0 0 0 0 0 0 0 0 0 1 1 1 0 0 0
Close and overlapping Anchorpoints are combined.
Anchorpoints with to low of a score are removed.
Bam file
***
0 0 0 0 1 0 0 0 0 0 0 0 0 0 1 1 1 0 0 0
Ap1
Ap2
6. Filter Anchorpoints that do not have:
6a. At least 20 reads between upstream and downstream combined
6b. A significant difference between upstream and downstream read-depth (p.val < 0.05, Binomial exact test)
6c. At least a 1.25-fold difference in read-depth upstream and downstream
7. Convert to scored breakpoint BED file.
Chromosome Start Stop UID Score
NC_001139.9 535644 535864 1 25
NC_001139.9 541575 541795 2 25
NC_001137.3 441531 441751 3 69
NC_001137.3 449208 449428 4 47
High Confidence Inverted Duplication Breakpoints

**Supplemental Methods Figure 1: Overview of Identification Pipeline**

**Sequencing failure causes difficulty in identification of CNV sequences between duplicated regions.**

Systemic, loci-specific, sequencing failure can have numerous effects to downstream analysis that can frustrate the resolution of CNV structure. Contemporary *de novo* assemblers differ in handling reads with long low-phred scoring regions. These may align all reads as best possible without regard to the phred score – this has the downside of generating many contigs separated solely by the mismatching nucleotides of the low-phred scoring region. Alternatively, reads with sufficiently long low-scoring regions will be discarded entirely. Finally, some assemblers may align reads while excluding, or discounting, low-quality regions – while preferable these often result in numerous contigs consisting of high-quality flanking regions with little high-confidence information pertaining to the inverted duplication junction.

Attempts to identify the sequence between inverted duplicated regions (ie. ‘spacers’) are also hampered by the sequencing failure inverted duplications can induce. Importantly, loss in sequencing accuracy can begin wherever a region has self-complementarity. Unfortunately, many sequence features that drive CNV formation are also self-complementary, making the identification of adjacent sequences difficult.

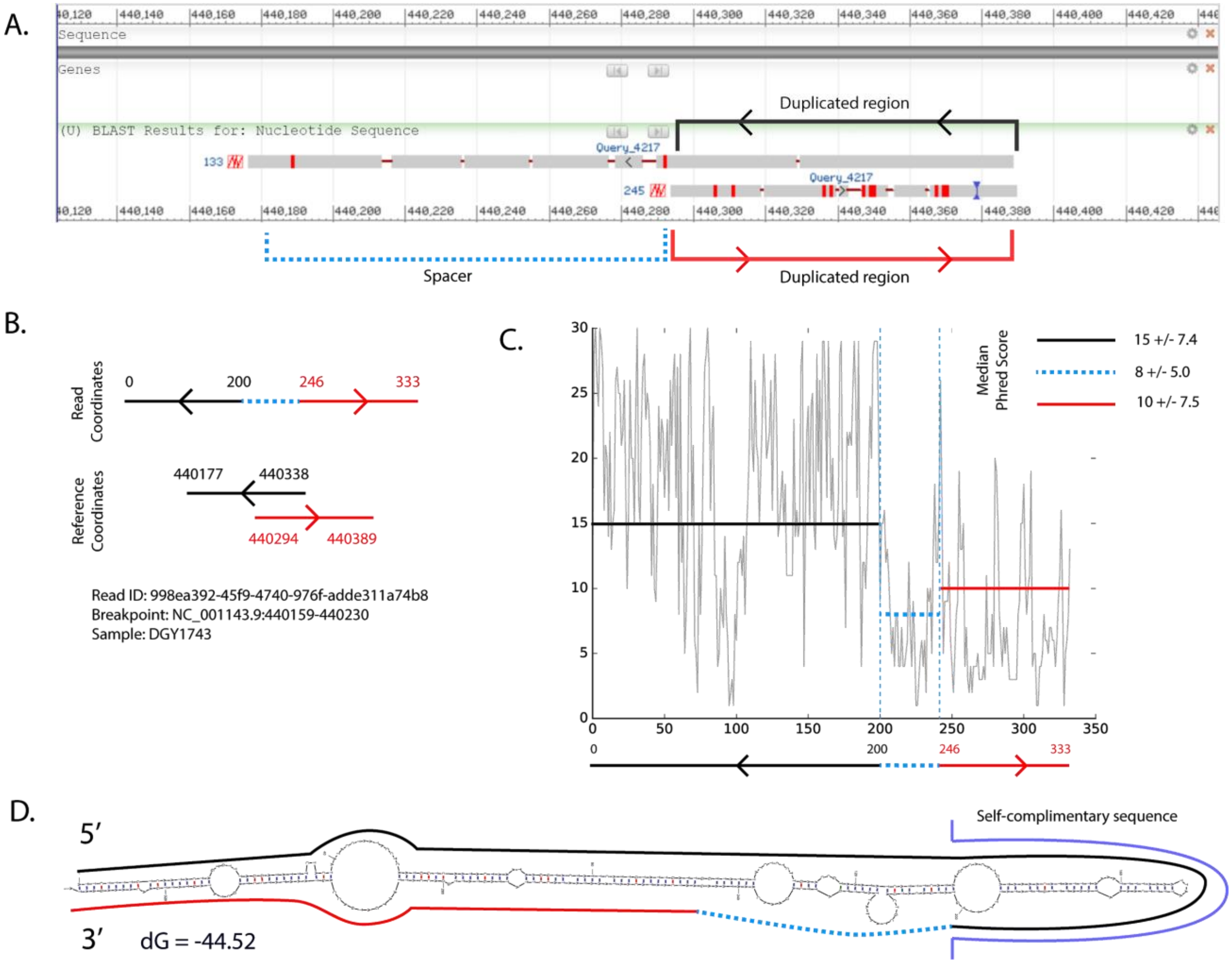


**Supplemental Methods Figure 2:**
The alignment of the region of a read involving both duplicated regions and the intervening spacer (**A**) (figure from BLAST). Schematic showing the Read and Reference coordinates as well as read identifiers (**B**). Plot of phred-score per nucleotide of the region with median phred-scores each region (**C**). Notably, the spacer region (dashed blue) has a low-phred score similar to that observed for the inverted duplicate regions (red). Diagram of predicted secondary structure showing the exogenous sequence features self-complementarity (**D)** (figure from Mfold (4)). Importantly, this secondary structure inhibits sequencing of the spacer region making the determination of the sequence difficult as single reads lack high accuracy and attempts at consensus construction fail due to highly divergent sequences.


4. M. Zuker. Mfold web server for nucleic acid folding and hybridization prediction.
Nucleic Acids Res. 31 (13), 3406-15, (2003)

**Determination of translocation rate**

Translocation rates of DNA through nanopores are highly variable, with substantial differences between pores, reads, and even the beginning and end of sequencing runs (5, 6).

Currently, ONT uses a combination of three data objects to perform base calling operations, these are stored in the fast5 file when the --fast5_output option is used with Guppy. 'Signal', also referred to as a squiggle, records the unscaled measurements of the current made by the sensors, there are approximately 4000 samples per second (6). 'Move' records the decisions of the base caller over whether or not a nucleotide has been read. This data object is a record of the number of bases which have been incremented per sample, that is a zero means that the base has not yet been fully sampled, a 1 indicates that a single base has been recorded, while a 2 means that two bases have been added at once. Signal and move are related to the sequenced bases by the additional 'stride' data object which is the nucleotides covered by each 'move' event.

To determine if the translocation rate changes within a low-phred scoring region we first map the coordinates of the aligned read into the signal map by using the nucleotide count provided by the move data object. We can then divide the number move events by the number of nucleotides in the reference coordinates.


5. Krause M, Niazi AM, Labun K, Torres Cleuren YN, Müller FS, Valen E. : alignment-free poly(A) length measurement for Oxford Nanopore RNA and DNA sequencing. RNA. 2019 Oct;25(10):1229–41.

6. Rang FJ, Kloosterman WP, de Ridder J. From squiggle to basepair: computational approaches for improving nanopore sequencing read accuracy. Genome Biol. 2018 Jul 13;19(1):90.

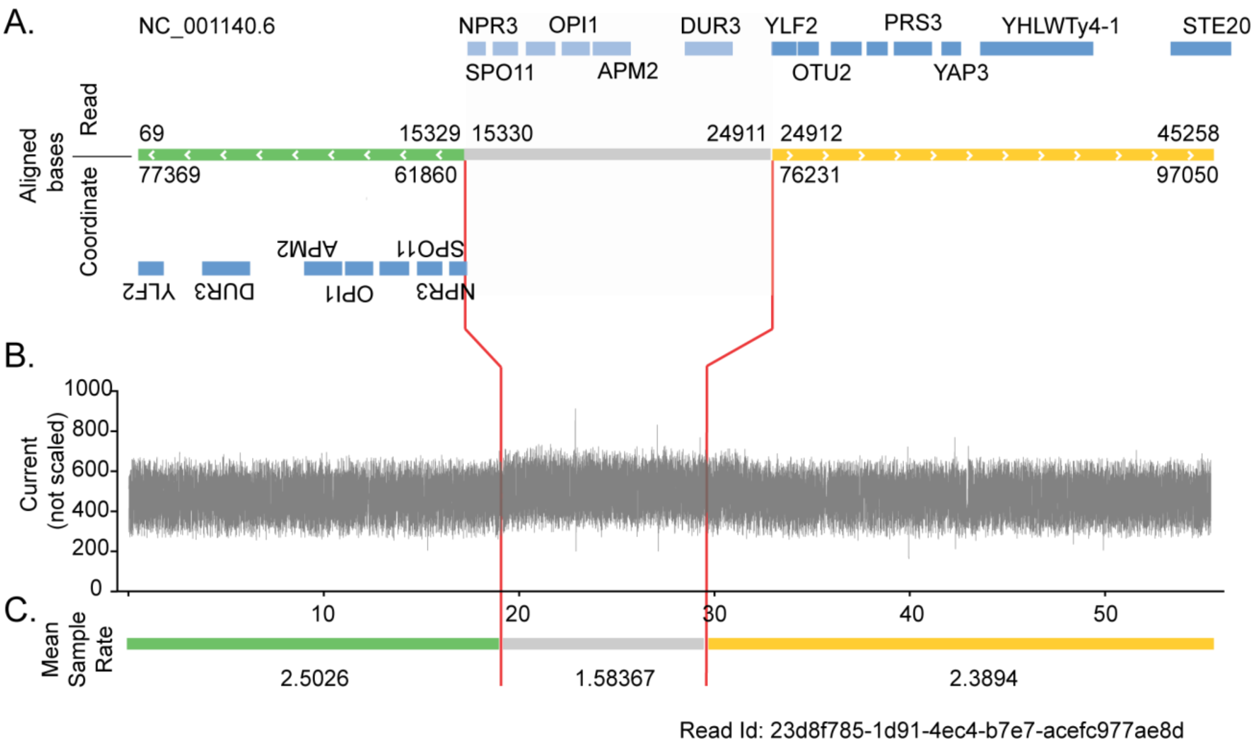


**Supplemental Methods Figure 3:**

Each aligned segment of the read (Green and Yellow bars) has Read specific nucleotides (Read, top) and Reference specific coordinates (Coordinate, bottom). Under ideal circumstances these two should be very near parity. Indeed, in this example the green and yellow segments are 98.4% and 97.7% identical, respectively, representing a loss of approximately 1 out of 50 bases.

However, for the unaligned segment (Grey bar) the expected region spanned is not equal to the number of sequenced bases, being only 66.7% the size expected, for a loss of about 1 in 3 bases.

To estimate the mean translocation rate for each segment we divide the number of samples for the segment by the number of coordinate bases the segment spans.
